# The *Microcephalin* Ancestral Allele in a Neanderthal Individual

**DOI:** 10.1371/journal.pone.0010648

**Published:** 2010-05-14

**Authors:** Martina Lari, Ermanno Rizzi, Lucio Milani, Giorgio Corti, Carlotta Balsamo, Stefania Vai, Giulio Catalano, Elena Pilli, Laura Longo, Silvana Condemi, Paolo Giunti, Catherine Hänni, Gianluca De Bellis, Ludovic Orlando, Guido Barbujani, David Caramelli

**Affiliations:** 1 Dipartimento di Biologia Evoluzionistica, Laboratori di Antropologia, Università di Firenze, Firenze, Italy; 2 Istituto di Tecnologie Biomediche, Consiglio Nazionale delle Ricerche (ITB-CNR), Segrate (MI), Italy; 3 Dipartimento di Scienze Ambientali “G. Sarfatti”, UR: Ecologia Preistorica, Università di Siena, Siena, Italy; 4 Museo di Storia Naturale di Verona - Sezione di Preistoria, Verona, Italy; 5 Laboratoire d'Anthropologie Bio-culturelle, UMR6578 Université de la Méditerranée -CNRS-EFS, Marseille, France; 6 Paleogenetics and Molecular Evolution, Institut de Génomique Fonctionnelle de Lyon, Université de Lyon, Université Lyon 1, CNRS, INRA, Ecole Normale Supérieure de Lyon, Lyon, France; 7 Dipartimento di Biologia ed Evoluzione, Università di Ferrara, Ferrara, Italy; University of Wisconsin, United States of America

## Abstract

**Background:**

The high frequency (around 0.70 worlwide) and the relatively young age (between 14,000 and 62,000 years) of a derived group of haplotypes, haplogroup D, at the *microcephalin* (*MCPH1*) locus led to the proposal that haplogroup D originated in a human lineage that separated from modern humans >1 million years ago, evolved under strong positive selection, and passed into the human gene pool by an episode of admixture circa 37,000 years ago. The geographic distribution of haplogroup D, with marked differences between Africa and Eurasia, suggested that the archaic human form admixing with anatomically modern humans might have been Neanderthal.

**Methodology/Principal Findings:**

Here we report the first PCR amplification and high- throughput sequencing of nuclear DNA at the *microcephalin* (*MCPH1*) locus from Neanderthal individual from Mezzena Rockshelter (Monti Lessini, Italy). We show that a well-preserved Neanderthal fossil dated at approximately 50,000 years B.P., was homozygous for the ancestral, non-D, allele. The high yield of Neanderthal mtDNA sequences of the studied specimen, the pattern of nucleotide misincorporation among sequences consistent with post-mortem DNA damage and an accurate control of the *MCPH1* alleles in all personnel that manipulated the sample, make it extremely unlikely that this result might reflect modern DNA contamination.

**Conclusions/Significance:**

The *MCPH1* genotype of the Monti Lessini (MLS) Neanderthal does not prove that there was no interbreeding between anatomically archaic and modern humans in Europe, but certainly shows that speculations on a possible Neanderthal origin of what is now the most common *MCPH1* haplogroup are not supported by empirical evidence from ancient DNA.

## Introduction

The gene *microcephalin* or *MCPH1* is a critical regulator of brain size. In humans, homozygosis for loss-of-function mutations in this gene causes a condition known as primary microcephaly, characterized by a severe reduction in brain volume (3- to 4-fold) but also by a retention of the overall neuroarchitecture, without evident defects outside of the brain [1]. The exact biochemical function of the *microcephalin* protein has yet to be elucidated, but it likely plays an essential role in promoting the proliferation of neural progenitor cells during neurogenesis [1].

*Microcephalin* has been proposed as the target of positive selection in the evolutionary lineage leading from ancestral primates to humans [2]. This observation, coupled with the fact that this gene is a critical regulator of brain size, suggests that the molecular evolution of *microcephalin* may have contributed to the phenotypic evolution of the human brain [2]. In a recent study, Evans *et al*. [3] found that a class of haplotypes at the locus, dubbed haplogroup D (all sharing a G37995C transversion in the coding region, that results in the substitution of an aspartate with a histidine), shows a recent coalescence age (point estimate  = 37,000 years) despite a very high worldwide frequency (0.70). There is also a marked difference between Africa (with low D frequencies) and Eurasia (with high D frequencies). Taken at face value, these findings would imply a rapid rise in the frequency of haplogroup D, so rapid indeed to suggest that positive selection, and not only drift or a demographic expansion, has affected the frequency of haplogroup D after the spread from Africa of early modern humans [3]. Evans and colleagues proposed that haplogroup D originated from a lineage separated from modern humans for 1.1 million years and introgressed into the human gene pool by 37,000 years ago, probably from a Neanderthal stock [4].

However, simulation approaches have shown that two key hypotheses of this model, namely positive selection and admixture, can be relaxed as long as Eurasia was settled from an African population that was both subdivided and under expansion [5]. Interestingly, variation in neurocranial geometry have recently suggested significant levels of geographic structure among early modern humans from Africa [6]. In addition, no direct empirical evidence supports a third key component of the model, ie. that Neanderthals carried alleles of the D haplogroup.

It is now possible to empirically test for a possible origin of haplogroup D in Neanderthals, by sequencing a fragment of the *microcephalin* locus overlapping the polymorphic position G37995C. The presence of *microcephalin* haplogroup D in Neanderthals would be the first molecular evidence of introgression between the two human forms, in sharp contrast with the results of mitochondrial studies suggesting that they never admixed (reviewed in Ref. 7). To test that hypothesis, we took advantage of a well preserved sample that already delivered authentic sequences from mitochondrial (hypervariable region I) and nuclear (*melanocortin 1 receptor*, *MC1R*) loci in order to reveal the Neanderthal genotype at the *microcephalin* locus [8], [9].

### Contaminations control

Contamination is a serious problem in ancient DNA studies, especially when human-like samples such as Neanderthals are analyzed [see e.g. 10], [ 11]. Contamination often occurs through direct handling and washing, presumably because DNA permeates through dentinal tubules into the pulp cavity (in teeth) and the Haversian system (in bone) [12], although possibly not reaching the osteocytes [13], [14]. In that context, the excavation in itself and the handling of the samples just afterwards appear crucially at risk [12], [15]. The Monti Lessini Neanderthal sample (MLS) consisted of small cranial fragments that did not allow extensive anthropological analysis and therefore were only superficially handled. Usually, post-mortem biochemical processes, such as strand fragmentation by hydrolysis, precludes or limits the analysis of nuclear DNA in ancient extracts. However, the MLS specimen is apparently in excellent state of preservation. In 2006 we sequenced its hypervariable region 1 (HVR-I) of the mitochondrial DNA (mtDNA) [8], and an average of 75% of endogenous sequences were retrieved among the sequences of the clones from independent PCR amplicons. Confirming that Neanderthal nuclear DNA can be successively recovered from sample with low level of modern human mtDNA contamination [16], we could retrieve an endogenous and informative fragment of the *MC1R* gene from this sample [9]. Therefore, we could reasonably expect high chances of success for genotyping a fragment of the *MCPH1* gene from the same DNA extract.

To determine the degree of modern human contamination in the aliquot of MLS DNA extract, we amplified and sequenced two diagnostic fragments of the mtDNA HVR-I between positions 16,109–16,191, and 16,220–16,282, as well as one position in the mitochondrial DNA (mtDNA) coding region (diagnostic A/C transversion 6,267) where there is a fixed difference between Neanderthals and modern humans [7], [17]. To ensure that the copy number of nuclear DNA was sufficient for amplifying nuclear Neanderthal DNA and to monitor potential nuclear DNA contamination, we additionally amplified a control position on the *lactase* gene [*LCT*] promoter (MCM6 locus). There are all reasons to believe that Neanderthals carried the ancestral allele, namely a C/T substitution at position -13910 with respect to the modern allele causing persistence of *lactase* enzyme production after waning, and thus of the ability to digest milk. Indeed, the allelic variant -13910T is estimated to have arisen less than 20,000 years ago in modern Europeans [18], [19], and to have risen to high frequencies under selective pressure in the areas of Europe where unfermented milk is a substantial component of diet [18], [20], [21], i.e. after the development of dairying technologies [22].

For each locus, we performed multiple PCRs, cloned and sequenced several clones from each PCR reaction (for a total number of 14 amplicons and 373 clones). To better test for the presence of different alleles at nuclear loci (due to either contamination from modern DNA or heterozygosis in the original sample) we sequenced two amplification products at high-coverage with the Roche/454 Life Science technology. In addition, to track down contaminations by the researchers involved in this study, all persons who manipulated the sample were genotyped for the relevant nuclear and mitochondrial DNA regions (in Florence, in a specific Forensic Laboratory). In this way we were able to control if any of the scientists' sequences matched those obtained in this study.

## Results

DNA was extracted from a skull fragment of MLS specimen in 2006, as reported in ref. 8 and 9. The extract was kept at – 20°C and a 10 µl aliquot was taken in 2008 for the analysis reported in this study.

Three different PCRs were performed for each diagnostic fragment on the mtDNA. All the amplifications except one (targeting the 6,267 transversion; Table 1) gave results. Each product was cloned and 20 clones sequenced. In the HVR-I region, 106 out of 120 clone sequences were Neanderthal-specific and showed the same motif previously reported for the MLS sample [8] (Table 1, Figure S1); the remaining 14 clones shared a modern human motif that matched with the profile obtained from one lab members, and actually the one (LL) who handled the samples most recently (Table 1, Figure S1). We targetted a DNA fragment exhibiting two fixed mutations between modern humans and Neanderthals. At the informative position 6,267 in the cytochrome oxidase I gene, 36 out of 40 clones presented a C. Moreover, all the 36 sequences with the 6,267C showed an A, a fixed characteristic of Neanderthal at position 6,261, whereas all modern humans have a C [7], [17] (Table 1, Figure S1). Therefore, globally, we recovered an average of 88% of Neanderthal-specific mtDNA sequences from this aliquot of MLS extract, a result in agreement with a previous analysis on the same extract (75%) [8] and reminiscent of what Lalueza-Fox *et al*. found preliminary to the study of AB0 variation in Neanderthals using the same markers (82–85%) [17]. Interestingly, nucleotide misincorporation among the Neanderthal mtDNA clone sequences shows a pattern typical of ancient templates (56,7% of overall mutations consist in type II transitions, ie GC→AT), in contrast to contaminants where no DNA damage induced mutations is observed (Table S4; chi square test p-values <2.6 10^−8^).

**Table 1:** Sequencing results for the amplifications of mtDNA fragments on MLS Neanderthal sample.

LOCUS	PCR	CLONES		454 FLX		
mtDNA		Ne	MH	Ne	MH	Ne%
16,109–16,191	1	20	0	-	-	100
	2	18	2	-	-	90
	3	20	0			100
16,220–16,282	1	17	3	-	-	85
	2	16	4	-	-	80
	3	15	5			75
6,267	1	no ampl.	no ampl.	-	-	
	2	17	3	-	-	85
	3	19	1			95
						88,75

‘CLONES’ means results from cloning and Sanger sequencing. ‘454 FLX’ means results from Roche/454 Life Science technology. (Ne) Neanderthal; (MH) Modern Human; (no ampl.) no amplification product obtained; (-) sequencing not attempted.

Nuclear internal control position in the *LCT* promoter was amplified in four independent PCRs. Three PCRs produced amplicons of the expected size, whereas one amplification gave no results (Table 2). Two amplicons were cloned and a total of 30 clones were sequenced. All the sequences showed a C at position -13,910, indicating that the MLS individual carried the ancestral allele of *LCT* promoter (Table 2, Figure S1). The third amplicon underwent emulsion PCR and subsequent pyrosequencing in the Roche/454 FLX machine; 45101 reads out 45120 had a C at position -13,910 while only 19 reads had a T (ie. 0.04%; Table 2, Table S1). Consistent with the mitochondrial information, nucleotide misincorporation among clone sequences consist all in GC→AT substitutions; in addition, no misincorporation is observed among the derived 454 reads as opposed to ancestral haplotypes (damage frequency per site  = 0.00036). All in all, this suggests that the templates analyzed are indeed ancient (Table S4).

**Table 2:** Sequencing results for the amplifications of nuclear loci on MLS Neanderthal sample.

LOCUS	PCR	CLONES		454 FLX		
nDNA		ancestral	derived	ancestral	derived	ancestral %
LCT (MCM6) -13910	1	30	0	-	-	100
	2	30	0	-	-	100
	3	no ampl.	no ampl.	-	-	-
	4	-	-	45101	19	99.96
MCPH1 37995	1	40	0	-	-	100
	2	no ampl.	no ampl.	-	-	-
	3	35	0	-	-	100
	4	40	0	-	-	100
	5	38	0	-	-	100
	6	no ampl.	no ampl.	-	-	-
	7	no ampl.	no ampl.	-	-	-
	8	-	-	12494	189	98.51

‘CLONES’ means results from cloning and Sanger sequencing. ‘454 FLX’ means results from Roche/454 Life Science technology. (no ampl.) no amplification product obtained; (-) sequencing not attempted.

The polymorphic position G37995C in *MCPH1* was checked by eight independent PCRs. The correct size amplicon was obtained in five PCRs, while three attempts gave no amplifications (Table 2). Four amplicons were cloned and a total of 153 clones were sequenced. A supplemental amplicon was sequenced in the Roche/454 FLX machine. None of the 153 clones sequenced presented the derived allele in position 37995 (Table 2, Figure S1) and nucleotide misincorporation consisted essentially in GC→AT transitions (85.71%; Table S4); in the 12683 sequences generated by the FLX machine the vast majority (12494) showed the ancestral allele while 189 (1.5%) presented the derived allele (Table 2, Table S2). In agreement to mitochondrial and *LCT* loci, only a single misincorporation is observed among the derived 454 reads as opposed to ancestral haplotypes; contrarily to patterns of taphonomic DNA degradation, it consists in a GC→TA transversion).

Consensus sequences of the three novel loci analysed in this study were deposited in Genbank (Accession numbers: GU191142, GU191143, GU191144).

All people who handled or worked on the MLS sample carried only the derived allele in both -13,910 *LCT* promoter and 37,995 *MCPH1* gene positions while mtDNA motifs for the region of interest are reported in SI (Table S3)

## Discussion

Our data show that the vast majority of clones amplified from the MLS Neanderthal extract contain the ancestral allele of *MCPH1*. This result (together with the high proportion of ancestral allele at *LCT* promoter -13910 control position) appears in agreement with a previous analysis of the same extract [8]. However, in another previous report [9] a moderate level (7–25%) of clones exhibited the presumed Neanderthal allele at the *MC1R* locus, suggesting that contaminant alleles could have outcompeted endogenous alleles (at the nuclear but not at the mitochondrial level). If true, the high frequencies of ancestral alleles found at *LCT* and *MCPH1* in the MLS extract would partially result from Neandertal and modern human templates. Importantly, the ancestral allele at *MCPH1* is found at significant frequencies among current European populations (22%, International HapMap Project, http://hapmap.ncbi.nlm.nih.gov/index.html.en, refSNP rs930557) and a significant fraction of the Southern European population is actually *lactase* deficient (eg. 64,3% in Northern Italy [18]). In addition, the ancestral allele at *LCT* has already been recovered from moderate (<17%) to maximal levels in non-human extracts, extraction and/or PCR controls, even though this allele is currently rare among human populations from the same geographic area [23]. We however do not believe that the *MCPH1* and *LCT* genotypes reported here result from contamination since several evidences support the authenticity of the results. First of all, high proportion of identical mtDNA sequences unequivocally Neanderthal-type were repeatedly obtained from the sample; even if there is not direct evidence that mtDNA and nuclear DNA contamination levels are equivalent (or not too different), low proportion of modern human mtDNA contaminants is a basic pre-requisite for nuclear loci investigation in Neanderthal samples [16], [17], [24]. Second, we produced for each positive amplification a large number of clone sequences (between 20 and 40), and we performed on selected amplicon ultra-deep sequencing by 454 technology to achieve still higher resolution in detecting both sporadic contamination and misincorporations due to damage. Third, the nucleotide misincorporation pattern in amplicon carrying the ancestral allele is effectively suggestive of ancient templates, opposite to amplicons with derived allele that do not exhibit this trend. Finally, none of the people who handled or worked on the MLS sample carried the ancestral allele at both -13,910 *LCT* promoter and 37,995 *MCPH1* gene positions (Table S3). Recently, new methods based on Primer Extension Capture have performed in limiting sequence retrieval from contaminant origins in ancient human extracts [25], [26]. Unfortunately, such methods cannot be used in the present case due to extract depletion, but, however, the most plausible hypothesis supported by all our findings is that the MLS individual was homozygous for the ancestral, non-D, *microcephalin* allele.

This result does not prove that there was no interbreeding between anatomically archaic and modern humans in Europe, but certainly shows that speculations on a possible Neanderthal origin of the most common *MCPH1* alleles [4] are currently unsupported by empirical evidence from ancient DNA. Of course, the possibility exists that *MCPH1* was polymorphic in Neanderthals, and that different individuals could carry a different allele. Support to this hypothesis can only be sought by studying other Neanderthal samples, and it is likely that it would receive shortly great attention due to the release of the complete Neanderthal genome. A statistically robust estimation of allele frequencies in Neanderthals may never be possible, but to maintain that the D haplogroup of *MCPH1* came from Neanderthals to modern humans it is crucial to find evidence of at least one haplogroup-D in Neanderthals. Therefore, at present we see no cogent reason to abandon simpler models in which the evolution of *microcephalin* diversity occurred entirely within populations of *Homo sapiens sapiens*.

The deep split in the gene genealogy described for *MCPH1* [4] has been observed at other loci as well, including *pyruvate dehydrogenase alpha 1* [27], *Dystrophin* [28], coagulation factor *FVII* [29], and two regions of chromosome X [30], [31]. These high levels of haplotype divergence, caused by several nucleotide substitutions between two major branches of the gene tree, have been interpreted as evidence for introgression followed by positive selection [32], but in fact that is not the only plausible explanation.

There is a broad agreement that the contribution of archaic *Homo* populations to the modern gene pool, if any, must have been very limited [33], [34]. Different lines of evidence concur to suggest that the dispersal of anatomically modern humans from Africa was accompanied by repeated founder effects [35]–[38]. If these founder effects were drastic, most or all gene genealogies should actually be shallow, and hence the occurrence of ancient splits would imply some degree of introgression from archaic human forms. However, different consequences would be expected if only mild founder effects occurred when anatomically modern humans moved out of Africa. Under these conditions, gene trees would have a strong random component, and a certain fraction thereof, even in the absence of selection, would show two highly divergent major lineages [39]. The likelihood of finding gene genealogies with a very old common ancestor and very differentiated lineages would be even higher if the source African population was subdivided and structured genetically before dispersal, which is what most studies clearly suggest [40]–[43]. These theoretical considerations are actually matched by consistent results in simulation studies [5], [34], [44] and by variation in neurocranial geometry, suggesting significant levels of geographic structure among early modern humans from Africa [6]. An empirical support for the possibility of mild founder effects leading to recent allelic divergence comes from the analysis of STR variation within the polymorphic, 900-kb inversion 17q21, also suggesting a recent origin (between 108,000 and 13,000 years) of the inversion [45], despite previous claims that its alleles may have diverged millions of years ago or had evolved under strong selective pressures [46].

In short, more data, in both modern populations and ancient samples, are needed to understand whether admixture between archaic and modern human forms was very limited or none, and we may not ever be able to test for minimal levels of genetic exchange [31]. Indeed, neutral alleles introduced at low frequency by introgression tend to rapidly disappear because of genetic drift, and so only if a new allele has some selective advantage do we seem to have a chance to retrieve any genetic evidence of introgression, even if introgression did occur [32]. On the other hand, some simulation studies suggest that massive introgression of neutral genes is to be expected when a group colonises a new territory, unless interbreeding is severely prevented between the two groups [47]. This implies that we would have a high probability to encounter Neanderthal alleles in the European gene pool, even at very low levels of introgression.

A sample of size one is clearly insufficient to reject any hypothesis, but within these strong restrictions, MLS is the first Neanderthal individual genotyped at the *MCPH1* locus and does not cluster within haplogroup-D. Along with the simulations of Ref. 5, this result indicates that at present we do not need either positive selection, or an origin outside African populations of *Homo sapiens sapiens*, to account for the observed patterns of modern *MCPH1* variation.

## Materials and Methods

### Sample

We analyzed one Neanderthal sample (MLS) from Mezzena Rockshelter in the Lessini Mountains (Verona, Italia) [48]. The DNA was extracted from a skull fragment in 2006, in a facility dedicated to ancient DNA study, and under the most stringent criteria for ancient DNA analysis, as described in ref. 7 and 8. Starting from a 2 gr fragment, two different powder aliquots, respectively of 1.5 and 0.5 gr, were extracted in two different ancient DNA laboratories and were used for the characterization of the entire mtDNA HVR-I and of a small fragment of the nuclear *MC1R* gene [8], [9]. In 2008 a 10 µl aliquot of the first extract, conserved at – 20°C, was used for the analysis reported in this study. No further amplifications were possible, due to the limited amount of material available.

### Ancient DNA amplification

Extracted DNA was diluted 1∶50 and 2–5 µl of this dilution were used in PCR reactions with the following profile: 94°C for 10 min and 50 cycles of denaturation (94°C for 45 sec), annealing (52–58°C, depending on primers sequences, for 1 min), and extension step (72°C for 1 min); the 50 µl reaction mix contained 2 units of AmpliTaq Gold polymerase, 1X reaction buffer (Applied Biosystems), 200 µM of each dNTP, 1.5 mM MgCl2, and 1 µM of each primer. Primers sequences and amplicons lengths for each amplified region were as follow:

#### Amplification of mtDNA Hypervariable Region I (HVR-I) diagnostic regions

Diagnostic fragments of mtDNA HVR-I were amplified with two different primers pairs: L16,109- TGTATTTCGTACATTACTGCCA/NH16,191-TGCTTGCTTGTAAGCATGGG and NL-16,220-CAAGCAAGCACAGCAATCA/H-16282-GGTAAGGGTGGGTAGGTTTG. Amplicons length was respectively 123 bp for the first primers couple and 101 bp for the second one.

#### Amplification of diagnostic transversion 6,267 of mt DNA

A 65 bp fragment of the mitochondrial cytochrome oxidase I gene was amplified using the primers pair as described in ref. 14.

#### Amplification of nuclear internal control (LCT promoter)

The C/T-13,910 polymorphic position in *lactase* gene promoter was checked by amplification of a 72 bp fragment in MCM6 locus with the primer pair -13910_F- CTGCGCTGGCAATACAGATA and -13910_R-CAAATGCAACCTAAGGAGGAG.

#### Amplification of *MCPH1* gene

A 76 bp fragment of *MCPH1* gene surrounding the polymorphic position G37995C was amplified using the primers pair G37995C_F- TTGCAAAGAAATATTGCAGGT and G37995C_R- CAAACGTCTCCTGAGACATACC.

For each locus between 3 and 8 PCR reactions were performed. Two negative controls were introduced in every PCR reactions. Amplification products of the correct size were gel isolated and purified.

### Sequence determination

The sequences of PCR products were determined by standard procedure of cloning and Sanger sequencing in Florence, or alternatively by emulsion PCR and subsequent pyrosequencing in Milan. Detailed procedures are reported below.

### Cloning and sequencing

PCR products were cloned using TOPO TA Cloning Kit (Invitrogen) according to the manufacturer's instructions. Screening of white recombinant colonies was accomplished by PCR, transferring the colonies into a 30 µl reaction mix (67 mM Tris HCl (pH 8.8), 2 mM MgCl2, 1 µM of each primer, 0.125 mM of each dNTP, 0.75 units of Taq Polymerase) containing M13 forward and reverse universal primers. After 5 min at 92°C, 30 cycles of PCR (30 s at 90°C, 1 min at 50°C, 1 min at 72°C) were carried out and clones with inserts of the expected size were identified by agarose gel electrophoresis. After purification of these PCR products with Microcon PCR devices (Amicon), a volume of 1.5 µl was cycle-sequenced following the BigDye Terminator kit (Applied Biosystems) supplier's instructions. The sequence was determined using an Applied BioSystems 3100 DNA sequencer. Between 20 and 40 clones for each amplification product were sequenced.

### 454 sample preparation and pyrosequencing of nuclear genes

Each PCR amplicon was checked by miniaturized capillary gel electrophoresis (Agilent 2100 Bioanalyzer, Agilent, Santa Clara, CA, USA), subsequently was purified (MinElute PCR Purification Kit, QIAGEN, Hilden, Germany). Amplicon were not fragmented and were processed to obtain the single-stranded template DNA (sstDNA) library as in Roche GS FLX library preparation protocol. Quality and quantity of sstDNA were checked by Agilent Bioanalyzer and RiboGreen RNA Quantitation Kit (Invitrogen, Carlsbad, CA). sstDNA library was bound onto DNA capture bead and amplified by emulsion PCR (emPCR) as reported in Roche GS FLX protocol. Positive DNA beads were prepared as in Roche GS FLX protocol, counted (Multisizer 3 Coulter Counter; Beckman Coulter, Fullerton, CA, USA) and sequenced by FLX Genome Sequencer (FLX Roche/454 LifeSciences). In the obtained reads primers sequences were masked and the resulting portion was mapped on the reference sequence (NCBI accession: BC030702, NG_008958) using the Amplicon Variant Analyzer application (AVA) by Roche, with default parameters. Finally, starting from the AVA multi-alignments, we generated the consensus sequences with a home-made Python script, which assigns for each position the most frequently base. Similarly, the pattern of nucleotide misincorporation has been automatically computed with a Perl script following [49] and considering that mutations present at the same of a given amplicon are not independent. The frequency of DNA damage per site per sequence (or read) is given after correcting the distribution of nucleotide misincorporations by the nucleotide composition of the amplicon [50].

### Tracking dawn modern contamination

Oral swabs were collected from the archaeologists, palaeontologists and laboratory researchers involved in the extraction and analysis of the Neanderthal remains. DNA was extracted using the Chelex method in a dedicated forensic lab in Florence. The same gene fragments analysed in the MLS Neanderthal sample were amplified with the same primers pairs used for the ancient sample except for mtDNA in which the entire HVR-I was determined with the primer pair L-15995 and H-16402. For each amplification product both strands were directly sequenced on an Applied Biosystems 3100 sequencer (ABI, Foster City, CA, USA).

## Supporting Information

Figure S1 DNA sequences from clones. For each locus the first line reports the reference sequence. Nucleotides identical to the reference sequence are indicated by dots. In the first column, clones are identified by PCR number and clone number for each PCR. HVR-I mtDNA clones are also aligned with all the homologous Neanderthal sequences deposited in Genbank, including a previous Lessini report; sample name and accession number are reported for each sequence.
(0.02 MB PDF)

Table S1 454 FLX sequencing results for LCT promoter locus. First column indicates base positions in the amplicons (primers regions excluded), second column base positions in the gene. Third column shows the base type for each position in the reference sequence. A_tot, C_tot, G_tot, T_tot and -_tot indicate the number of reads with respectively A,C,G, T and no base in each position. The last column shows the total number of reads for each position. Positions of interest are highlighted in bold.
(0.17 MB DOC)

Table S2 454 FLX sequencing results for mcph1 37995 locus. First column indicates base positions in the amplicons (primers regions excluded), second column base positions in the gene. Third column shows the base type for each position in the reference sequence. A_tot, C_tot, G_tot, T_tot and -_tot indicate the number of reads with respectively A,C,G, T and no base in each position. The last column shows the total number of reads for each position. Positions of interest are highlighted in bold.
(0.13 MB DOC)

Table S3 Mitochondrial HVR1 variation in the researchers that have been in physical contact with the samples.
(0.03 MB DOC)

Table S4 Nucleotide misincorporations among clone sequences and 454 reads. The distributions of each possible misincorporation are reported. %P(TsI), %P(TsII) and %P(Tv) refer to the observed frequencies (%) of type I and type II transitions and transversion per site (ignoring indels) and corrected for nucleotide composition. For a given locus, the probability values reported correspond to weight averages over the different amplicons.
(0.09 MB DOC)
