# Supplementary material for: The Microcephalin Ancestral Allele in a Neanderthal Individual
Source: PLoS One. 2010 May 14;5(5):e10648. doi: 10.1371/journal.pone.0010648 (PMC2871044; doi:10.1371/journal.pone.0010648)
Supplement: Figure S1 — DNA sequences from clones. For each locus the first line reports the reference sequence. Nucleotides identical to the reference sequence are indicated by dots. In the first column, clones are identified by PCR number and clone number for each PCR. HVR-I mtDNA clones are also aligned with all the homologous Neanderthal sequences deposited in Genbank, including a previous Lessini report; sample name and accession number are reported for each sequence. (0.02 MB PDF) [file pone.0010648.s001.pdf]

mtDNA HVR-I 16,109-16,191

```
CRS       TGTATTTCGTACATTACTGCCAGCCACCATGAATATTGTACGGTACCATAAATACTTGACCACCTGTAGTACATAAAAACCCAATCCACATCAAAACCCCCTCCCCATGCTTACAAGCAAGTA

Mezmaiskaya FM865411, AF254446  ...................A.........T........T.......A............T............CC....C.
Teshik-Tash EU078679            --------------------.........T........T.......A............T.............C...TC.
Feldhofer FM865407              ...................A.........T........T.....C..............T.............C....C.
Feldhofer AY149291              ...................A.........T........T.....C..............T............CC....C.
Sidron DQ859014                 ...................A.........T........T.....C..............T.........C..CC....C.
Sidron FM865409                 .............................T........T.....C..............T.............C....C.
Sidron FM866397                 ...................A.........T........T.....C..............T.............C....C.
Vindija AF282971, AM948965      ...................A.........T........T.....C..............T............CC....C.
Vindija FM865410                ...................A.........T........T.....C..............T.............C....C.
Okladnikov EU078680             ...................A.........T........T....................T..C..........C......
Lessini DQ836132                ...................A.........T........T....................T............CC....C.

PCR1.CLO1 TGTATTTCGTACATTACTGCCA...................A.........T........T....................T............CC....C.CCCATGCTTACAAGCAAGCA
PCR1.CLO2               L16,109 ...................A.........T........T....................T............CC....C. NH16,191
PCR1.CLO3                       ...................A.........T........T....................T............CC....C.
PCR1.CLO4                       ...................A.........T........T................T...T............CC....C.
PCR1.CLO5                       ....TT.............A.........T........T....................T............CC....C.
PCR1.CLO6                       ...................A.....G...T........T....................T............CC....C.
PCR1.CLO7                       ...................A.........T........T....................T............CC....C.
PCR1.CLO8                       ...................A.........T........T....................T............CC....C.
PCR1.CLO9                       ...................A.........T........T....................T............CC....C.
PCR1.CLO10                      ...................A.........T........T....................T............CC....C.
PCR1.CLO11                      ...................A.........T........T....................T............CC....C.
PCR1.CLO12                      ....TT.............A.........T........T....................T............CC....C.
PCR1.CLO13                      ...................A.........T........T....................T............CC....C.
PCR1.CLO14                      ...................A.....G...T........T....................T............CC....C.
PCR1.CLO15                      ...................A.........T........T....................T............CC....C.
PCR1.CLO16                      ...................A.........T........T....................T............CC....C.
PCR1.CLO17                      ...................A.........T........T....................T............CC....C.
PCR1.CLO18                      ...................A.........T........T....................T............CC....C.
PCR1.CLO19                      ...................A.........T........T....................T............CC....C.
PCR1.CLO20                      ...................A.........T........T....................T............CC....C.

PCR2.CLO1 TGTATTTCGTACATTACTGCCA...................A.........T........T....................T............CC....C.CCCATGCTTACAAGCAAGCA
PCR2.CLO2               L16,109 ...................A.........T........T....................T............CC....C. NH16,191
PCR2.CLO3                       ...................A.........T........T....................T............CC....C.
PCR2.CLO4                       ...................A.........T........T....................T............CC....C.
PCR2.CLO5                       ....TT.............A.........T........T....................T............CC....C.
PCR2.CLO6                       ....TT.............A.........T........T....................T............CC....C.
PCR2.CLO7                       ...................A.........T........T....................T............CC....C.
PCR2.CLO8                       ...................A.........T........T....................T............CC....C.
PCR2.CLO9                       ...................A.........T........T....................T............CC....C.
PCR2.CLO10                      ...................A.........T........T....................T............CC....C.
PCR2.CLO11                      ...................A.........T........T....................T............CC....C.
PCR2.CLO12                      ...................A.........T........T..C.................T............CC....C.
PCR2.CLO13                      ...................A.........T........T....................T............CC....C.
PCR2.CLO14                      ...................A.........T........T....................T............CC....C.
PCR2.CLO15                      ...................A.........T........T....................T............CC....C.
PCR2.CLO16                      ...................A.........T........T....................T............CC....C.
PCR2.CLO17                      ...................A.........T........T....................T............CC....C.
PCR2.CLO18                      ...................A.........T........T....................T............CC....C.
PCR2.CLO19                      ..................................................................T.............
PCR2.CLO20                      ..................................................................T.............

PCR3.CLO1 TGTATTTCGTACATTACTGCCA...................A.........T........T....................T............CC....C.CCCATGCTTACAAGCAAGCA
PCR3.CLO2               L16,109 ...................A.........T........T....................T............CC....C. NH16,191
PCR3.CLO3                       ...................A.........T........T....................T............CC....C.
PCR3.CLO4                       ...................A.........T........T....................T............CC....C.
PCR3.CLO5                       ...................A.........T........T....................T............CC....C.
PCR3.CLO6                       ...................A.........T........T....................T............CC....C.
PCR3.CLO7                       ...................A.........T........T....................T............CC....C.
PCR3.CLO8                       ...................A.........T........T....................T............CC....C.
PCR3.CLO9                       ...................A.........T........T....................T............CC....C.
PCR3.CLO10                      ...................A.........T........T....................T............CC....C.
PCR3.CLO11                      ...................A.........T........T....................T..T.........CC....C.
PCR3.CLO12                      ...................A.........T........T....................T............CC....C.
PCR3.CLO13                      ...................A.........T........T....................T............CC....C.
PCR3.CLO14                      ...................A.........T........T....................T............CC....C.
PCR3.CLO15                      ...................A.........T........T.......C............T............CC....C.
PCR3.CLO16                      ...................A.........T........T....................T............CC....C.
PCR3.CLO17                      ...................A.........T........T....................T............CC....C.
PCR3.CLO18                      ...................A.........T........T....................T............CC....C.
PCR3.CLO19                      ...................A.........T........T....................T............CC....C.
PCR3.CLO20                      ...................A.........T........T....................T............CC....C.
```

mtDNA HVR-I 16,220-16,282

```
CRS         CAAGCAAGCACAGCAATCAACCCTCAACTATCACACATCAACTGCAACTCCAAAGCCACCCCT-CACCCACTAGGATACCAACAAACCTACCCACCCTTAAC

Mezmaiskaya FM865411, AF254446 ...T......G...T.........A...........A.....T.A..............T...
Teshik-Tash EU078679           ...T......G...T.......TCA...........A.....T.A..T.......A...T...
Feldhofer FM865407             ...T......G...T.........A...........A.G...T.A..............T...
Feldhofer AY149291             ...T......G...T.........A...........A.....T.A..............T...
Sidron DQ859014                ...T......G...T.........A...........A.G...T.A..............T...
Sidron FM865409                ...T......G...T.........A...........A.G...T.A..............T...
Sidron FM866397                ...T......G...T.........A...........A.G...T.A..............T...
Vindija AF282971, AM948965     ...T......G...T.........A...........A.G...T.A..............T...
Vindija FM865410               ...T......G...T.........A...........A.G...T.A..............T...
Okladnikov EU078680            ...T......G...T.........A...........A.....TCA..............T...
Scladina DQ464008              ...T......G...T........CA...........A.....T.A..T...........T...
Lessini DQ836132               ...T......G...T.........A...........A.G...T.A..................

PCR1.CLO1   CAAGCAAGCACAGCAATCA...T......G...T.........A...........A.G...T.A..................CAAACCTACCCACCCTTACC
PCR1.CLO2             NL16,220 ...T......G...T.........A...........A.G...T.A..TT.............. H16,282
PCR1.CLO3                      ...T......G...T.........A...........A.G...T.A..................
PCR1.CLO4                      ...T......G...T.........A...........A.G...T.A..................
PCR1.CLO5                      ...T......G...T.........A...........A.G...T.A..................
PCR1.CLO6                      ...T......G...T.........A...........A.G...T.A..................
PCR1.CLO7                      ...T......G...T.........A...........A.G...T.A..................
PCR1.CLO8                      ...T......G...T.........A...........A.G...T.A..................
PCR1.CLO9                      ...T......G...T.........A....C......A.G...T.A..................
PCR1.CLO10                     ...T......G...T.........A...........A.G...T.A..................
PCR1.CLO11                     ...T......G...T.........A...........A.G...T.A..................
PCR1.CLO12                     ...T......G...T.........A...........A.G...T.A..................
PCR1.CLO13                     ...T......G...T.........A...........A.G...T.A..................
PCR1.CLO14                     ...T......G...T.........A...........A.G...T.A..................
PCR1.CLO15                     ...T......G...T.........A...........A.G...T.A..................
PCR1.CLO16                     ...T......G...T.........A...........A.G...T.A..................
PCR1.CLO17                     ...T......G...T.........A...........A.G...T.A..................
PCR1.CLO18                     ............................................-..........A.......
PCR1.CLO19                     ............................................-..........A.......
PCR1.CLO20                     ............................................-..........A.......

PCR2.CLO1   CAAGCAAGCACAGCAATCA...T......G...T.........A...........A.G...T.A..................CAAACCTACCCACCCTTACC
PCR2.CLO2             NL16,220 ...T......G...T.........A...........A.G...T.A.................. H16,282
PCR2.CLO3                      ...T......G...T.........A...........A.G...T.A..................
PCR2.CLO4                      ...T......G...T.........A...........A.G...T.A..................
PCR2.CLO5                      ...T......G...T.........A...........A.G...T.A..................
PCR2.CLO6                      ...T......G...T.........A...........A.G...T.A................T.
PCR2.CLO7                      ...T......G...T.....G...A...........A.G...T.A..................
PCR2.CLO8                      ...T......G...T......G..A...........A.G...T.A..................
PCR2.CLO9                      ...T......G...T.........A...........A.G...T.A..................
PCR2.CLO10                     ...T......G...T.........A...........A.G...T.A..................
PCR2.CLO11                     ...T......G...T.........A...........A.G...T.A..................
PCR2.CLO12                     ...T......G...T.........A...........A.G...T.A..................
PCR2.CLO13                     ...T......G...T.........A...........A.G...T.A..TT..............
PCR2.CLO14                     ...T......G...T.........A...........A.G...T.A..................
PCR2.CLO15                     ...T......G...T.........A...........A.G...T.A..................
PCR2.CLO16                     ...T......G...T.........A...........A.G...T.A..................
PCR2.CLO17                     ............................................-..........A.......
PCR2.CLO18                     ............................................-..........A.......
PCR2.CLO19                     ............................................-..........A.......
PCR2.CLO20                     ............................................-..........A.......

PCR3.CLO1   CAAGCAAGCACAGCAATCA...T......G...T.........A...........A.G...T.A..................CAAACCTACCCACCCTTACC
PCR3.CLO2             NL16,220 ...T......G...T.........A...........A.G...T.A.................. H16,282
PCR3.CLO3                      ...T......G...T.........A...........A.G...T.A..................
PCR3.CLO4                      ...T......G...T.........A......G....A.G...T.A..................
PCR3.CLO5                      ...T......G...T.........A...........A.G...T.A..................
PCR3.CLO6                      ...T......G...T.........A...........A.G...T.A..................
PCR3.CLO7                      ...T......G...T.........A...........A.G...T.A..................
PCR3.CLO8                      ...T......G...T.........A...........A.G...T.A..................
PCR3.CLO9                      ...T..G...G...T.........A...........A.G...T.A..................
PCR3.CLO10                     ...T......G...T.........A...........A.G...T.A..................
PCR3.CLO11                     ...T......G...T.........A...........A.G...T.A..................
PCR3.CLO12                     ...T......G...T.........A...........A.G...T.A..................
PCR3.CLO13                     ...T......G...T.........A...........A.G...T.A..................
PCR3.CLO14                     ...T......G...T.........A...........A.G...T.A..................
PCR3.CLO15                     ...T......G...T.........A...........A.G...T.A..................
PCR3.CLO16                     ............................................-..........A.......
PCR3.CLO17                     ............................................-..........A.......
PCR3.CLO18                     ............................................-..........A.......
PCR3.CLO19                     ............................................-..........A.......
PCR3.CLO20                     ............................................-..........A.......
```

mtDNA 6,267

```
CRS             TGCTCGCATCTGCTATAGTGGAGGCCGGAGCAGGAACAGGTTGAACAGTCTACCCTCCCTTAGCA
PCR2.CLO1       TGCTCGCATCTGCTATAGTG..A.....C................CAGTCTACCCTCCCTTAGCA
PCR2.CLO2                           ..A.....C................
PCR2.CLO3                           ..A.TT..C................
PCR2.CLO4                           ..A.....C................
PCR2.CLO5                           ..A.....C................
PCR2.CLO6                           ..A.....C................
PCR2.CLO7                           ..A.....C................
PCR2.CLO8                           ..A.....C..........A.....
PCR2.CLO9                           ..A.....C................
PCR2.CLO10                          ..A.....C................
PCR2.CLO11                          ..A.....C................
PCR2.CLO12                          ..A.....C................
PCR2.CLO13                          ..A.....C................
PCR2.CLO14                          ..A.....C................
PCR2.CLO15                          ..A.....C................
PCR2.CLO16                          ..A.....C................
PCR2.CLO17                          ..A.....C................
PCR2.CLO18                          .........................
PCR2.CLO19                          .........................
PCR2.CLO20                          .........................

PCR3.CLO1       TGCTCGCATCTGCTATAGTG..A.....C................CAGTCTACCCTCCCTTAGCA
PCR3.CLO2                           ..A.....C................
PCR3.CLO3                           ..A.....C................
PCR3.CLO4                           ..A..T..C................
PCR3.CLO5                           ..A.....C................
PCR3.CLO6                           ..A.....C............A...
PCR3.CLO7                           ..A.....C................
PCR3.CLO8                           ..A.....C................
PCR3.CLO9                           ..A.....C................
PCR3.CLO10                          ..A.....C................
PCR3.CLO11                          ..A.....C................
PCR3.CLO12                          ..A.....C................
PCR3.CLO13                          ..A.....C................
PCR3.CLO14                          ..A.....C................
PCR3.CLO15                          ..A.....C................
PCR3.CLO16                          ..A.....C................
PCR3.CLO17                          ..A.....C................
PCR3.CLO18                          ..A.....C................
PCR3.CLO19                          ..A.....C................
PCR2.CLO18                          .........................
PCR2.CLO19                          .........................
PCR2.CLO20                          .........................
PCR3.CLO20                          .........................
```

LCT promoter (MCM6)

```
ref_NG_008958    CTGCGCTGGCAATACAGATAAGATAATGTAGCCCCTGGCCTCAAAGGAACTCTCCTCCTTAGGTTGCATTTG

PCR1.CLO1        CTGCGCTGGCAATACAGATA...............................CTCCTCCTTAGGTTGCATTTG
PCR1.CLO2        13910_F             ...............................                13910R
PCR1.CLO3                            ...............................
PCR1.CLO4                            ...............................
PCR1.CLO5                            ...............................
PCR1.CLO6                            ...............................
PCR1.CLO7                            ...............................
PCR1.CLO8                            ...............................
PCR1.CLO9                            ...............................
PCR1.CLO10                           ...............................
PCR1.CLO11                           ...............................
PCR1.CLO12                           ...............................
PCR1.CLO13                           ...............................
PCR1.CLO14                           ...............................
PCR1.CLO15                           ...............................
PCR1.CLO16                           ...............................
PCR1.CLO17                           ...............................
PCR1.CLO18                           ...............................
PCR1.CLO19                           .A.............................
PCR1.CLO20                           ...............................
PCR1.CLO21                           ...............................
PCR1.CLO22                           ...............................
PCR1.CLO23                           ...............................
PCR1.CLO24                           ...............................
PCR1.CLO25                           ...............................
PCR1.CLO26                           ...............................
PCR1.CLO27                           ...............................
PCR1.CLO28                           ...........TT..................
PCR1.CLO29                           ...............................
PCR1.CLO30                           ...............................

PCR2.CLO1        CTGCGCTGGCAATACAGATA...............................CTCCTCCTTAGGTTGCATTTG
PCR2.CLO2        13910_F             ...............................                13910R
PCR2.CLO3                            ...............................
PCR2.CLO4                            ...............................
PCR2.CLO5                            ...............................
PCR2.CLO6                            ...............................
PCR2.CLO7                            ...............................
PCR2.CLO8                            ...............................
PCR2.CLO9                            ...............................
PCR2.CLO10                           ...............................
PCR2.CLO11                           ...............................
PCR2.CLO12                           ...............................
PCR2.CLO13                           ...............................
PCR2.CLO14                           ...............................
PCR2.CLO15                           ...............................
PCR2.CLO16                           ...............................
PCR2.CLO17                           ...............................
PCR2.CLO18                           ...............................
PCR2.CLO19                           ...............................
PCR2.CLO20                           ...............................
PCR2.CLO21                           ...............................
PCR2.CLO22                           ...............................
PCR2.CLO23                           ...............................
PCR2.CLO24                           ...............................
PCR2.CLO25                           ...............................
PCR2.CLO26                           ...............................
PCR2.CLO27                           ...............................
PCR2.CLO28                           ...............................
PCR2.CLO29                           ...............................
PCR2.CLO30                           ...............................
```

MCPH1

```
ref_BC030702      TTGCAAAGAAATATTGCAGGTAAAGTAGTCACCCCTCACCAAAAGCAGGCTGCAGGTATGTCTCAGGAGACGTTTG

PCR1.CLO1         TTGCAAAGAAATATTGCAGGT...............G.................GGTATGTCTCAGGAGACGTTTG
PCR1.CLO2         G37995C_F            ...............G.................           G37995C_R
PCR1.CLO3                              ...............G.................
PCR1.CLO4                              ...............G.................
PCR1.CLO5                              ...............G.................
PCR1.CLO6                              ...............G.................
PCR1.CLO7                              ...............G.................
PCR1.CLO8                              ...............G.................
PCR1.CLO9                              ...............G.................
PCR1.CLO10                             ...............G.................
PCR1.CLO11                             ...............G.................
PCR1.CLO12                             ...............G.................
PCR1.CLO13                             ...............G.................
PCR1.CLO14                             ...............G.................
PCR1.CLO15                             ...............G.................
PCR1.CLO16                             ...............G.................
PCR1.CLO17                             ...............G.................
PCR1.CLO18                             ...............G.................
PCR1.CLO19                             ...............G.................
PCR1.CLO20                             ...............G.................
PCR1.CLO21                             ...............G.................
PCR1.CLO22                             ...............G.................
PCR1.CLO23                             ...............G.................
PCR1.CLO24                             ...............G.................
PCR1.CLO25                             ...............G.................
PCR1.CLO26                             ...............G.................
PCR1.CLO27                             ...............G.................
PCR1.CLO28                             ...............G.................
PCR1.CLO29                             ...............G.................
PCR1.CLO30                             ...............G.................
PCR1.CLO31                             ...............G.................
PCR1.CLO32                             ...............G.................
PCR1.CLO33                             ...............G.................
PCR1.CLO34                             ...............G.................
PCR1.CLO35                             ...............G.................
PCR1.CLO36                             ...............G.................
PCR1.CLO37                             ..........TT...G.................
PCR1.CLO38                             ...............G.................
PCR1.CLO39                             ...............G.................
PCR1.CLO40                             ...............G.................

PCR3.CLO1         TTGCAAAGAAATATTGCAGGT...............G.....T...........GGTATGTCTCAGGAGACGTTTG
PCR3.CLO2         G37995C_F            ...............G.................           G37995C_R
PCR3.CLO3                              ...............G.................
PCR3.CLO4                              ...............G.................
PCR3.CLO5                              ...............G.................
PCR3.CLO6                              ...............G.................
PCR3.CLO7                              ...............G.................
PCR3.CLO8                              ...............G.................
PCR3.CLO9                              ...............G.................
PCR3.CLO10                             ...............G.................
PCR3.CLO11                             ...............G.................
PCR3.CLO12                             ...............G.................
PCR3.CLO13                             ...............G.................
PCR3.CLO14                             ...............G.................
PCR3.CLO15                             ...............G.................
PCR3.CLO16                             ...............G.................
PCR3.CLO17                             ...............G.................
PCR3.CLO18                             ...............G.......A.........
PCR3.CLO19                             ...............G.................
PCR3.CLO20                             ...............G.................
PCR3.CLO21                             ...............G.................
PCR3.CLO22                             ...............G.................
PCR3.CLO23                             ...............G.................
PCR3.CLO24                             ...............G.................
PCR3.CLO25                             ...............G.................
PCR3.CLO26                             ...............G.................
PCR3.CLO27                             ...............G.................
PCR3.CLO28                             ...............G.................
PCR3.CLO29                             ...............G.................
PCR3.CLO30                             ...............G.................
PCR3.CLO31                             ...............G.................
PCR3.CLO32                             ...............G.................
PCR3.CLO33                             ...............G.................
PCR3.CLO34                             ...............G.................
PCR3.CLO35                             ...............G.................

PCR4.CLO1         TTGCAAAGAAATATTGCAGGT...............G.................GGTATGTCTCAGGAGACGTTTG
PCR4.CLO2         G37995C_F            ...............G.................           G37995C_R
PCR4.CLO3                              ...............G.................
PCR4.CLO4                              ...............G.................
PCR4.CLO5                              ...............G.................
PCR4.CLO6                              ...............G.................
PCR4.CLO7                              ...............G.................
PCR4.CLO8                              ...............G.................
PCR4.CLO9                              ...............G.................
PCR4.CLO10                             ...............G.................
PCR4.CLO11                             ...............G.................
PCR4.CLO12                             ...............G.................
PCR4.CLO13                             ...............G.................
PCR4.CLO14                             ...............G.................
PCR4.CLO15                             ...............G.................
PCR4.CLO16                             ...............G.................
PCR4.CLO17                             ...............G.................
PCR4.CLO18                             ...............G.................
PCR4.CLO19                             ...............G.................
PCR4.CLO20                             ...............G.................
PCR4.CLO21                             ...............G.................
PCR4.CLO22                             ...............G.................
PCR4.CLO23                             ...............G.................
PCR4.CLO24                             ...............G.................
PCR4.CLO25                             ...............G.................
PCR4.CLO26                             ...............G.................
PCR4.CLO27                             ...............G........T........
PCR4.CLO28                             ...............G.................
PCR4.CLO29                             ...............G.................
PCR4.CLO30                             ...............G.................
PCR4.CLO31                             ...............G.................
PCR4.CLO32                             ...............G.................
PCR4.CLO33                             ...............G.................
PCR4.CLO34                             ...............G.................
PCR4.CLO35                             ...............G.................
PCR4.CLO36                             ...............G.................
PCR4.CLO37                             ..........TT...G.................
PCR4.CLO38                             ...............G.................
PCR4.CLO39                             ...............G.................
PCR4.CLO40                             ...............G.................

PCR5.CLO1         TTGCAAAGAAATATTGCAGGT...............G.................GGTATGTCTCAGGAGACGTTTG
PCR5.CLO2         G37995C_F            ...............G.................           G37995C_R
PCR5.CLO3                              ...............G.................
PCR5.CLO4                              ...............G.................
PCR5.CLO5                              ...............G.................
PCR5.CLO6                              ...............G.................
PCR5.CLO7                              ...............G.................
PCR5.CLO8                              ...............G.................
PCR5.CLO9                              ...............G.................
PCR5.CLO10                             ...............G.................
PCR5.CLO11                             ...............G.................
PCR5.CLO12                             ...............G.................
PCR5.CLO13                             ...............G.................
PCR5.CLO14                             ...............G.................
PCR5.CLO15                             ...............G.................
PCR5.CLO16                             ...............G.................
PCR5.CLO17                             ...............G.................
PCR5.CLO18                             ...............G.................
PCR5.CLO19                             ...............G.................
PCR5.CLO20                             ...............G.................
PCR5.CLO21                             ...............G.................
PCR5.CLO22                             ...............G.................
PCR5.CLO23                             ...............G.................
PCR5.CLO24                             ...............G.................
PCR5.CLO25                             ...............G.................
PCR5.CLO26                             ...............G.................
PCR5.CLO27                             ...............G.................
PCR5.CLO28                             ...............G.................
PCR5.CLO29                             ...............G.................
PCR5.CLO30                             ...............G.................
PCR5.CLO31                             ...............G.................
PCR5.CLO32                             ...............G.................
PCR5.CLO33                             ...............G.................
PCR5.CLO34                             ...............G.................
PCR5.CLO35                             ...............G.................
PCR5.CLO36                             ...............G.................
PCR5.CLO37                             ...............G.................
PCR5.CLO38                             ...............G.................
```
