# Supplementary material for: The Microcephalin Ancestral Allele in a Neanderthal Individual
Source: PLoS One. 2010 May 14;5(5):e10648. doi: 10.1371/journal.pone.0010648 (PMC2871044; doi:10.1371/journal.pone.0010648)
Supplement: Table S1 — 454 FLX sequencing results for LCT promoter locus. First column indicates base positions in the amplicons (primers regions excluded), second column base positions in the gene. Third column shows the base type for each position in the reference sequence. A_tot, C_tot, G_tot, T_tot and -_tot indicate the number of reads with respectively A,C,G, T and no base in each position. The last column shows the total number of reads for each position. Positions of interest are highlighted in bold. (0.17 MB DOC) [file pone.0010648.s002.doc]

| **LCT promoter (MCM6)** | | |  |  |  |  |  |  |
| --- | --- | --- | --- | --- | --- | --- | --- | --- |
|  |  |  |  |  |  |  |  |  |
| **#Pos** | **Pos_ref** | **Base_ref** | **A_tot** | **C_tot** | **G_tot** | **T_tot** | **-_tot** | **Tot** |
| 1 | -13921 | A | 45106 | 0 | 1 | 0 | 0 | 45107 |
| 1.1 | -13921.1 | - | 1 | 0 | 0 | 0 | 45106 | 45107 |
| 2 | -13920 | G | 11 | 0 | 45100 | 1 | 2 | 45114 |
| 2.1 | -13920.1 | - | 0 | 0 | 2 | 1 | 45111 | 45114 |
| 3 | -13919 | A | 45110 | 0 | 2 | 0 | 0 | 45112 |
| 3.1 | -13919.1 | - | 2 | 0 | 3 | 0 | 45109 | 45114 |
| 4 | -13918 | T | 0 | 1 | 0 | 45113 | 0 | 45114 |
| 5 | -13917 | A | 45115 | 0 | 1 | 0 | 0 | 45116 |
| 6 | -13916 | A | 45116 | 0 | 0 | 0 | 0 | 45116 |
| 6.1 | -13916.1 | - | 6 | 6 | 1 | 0 | 45103 | 45116 |
| 7 | -13915 | T | 0 | 4 | 0 | 45111 | 1 | 45116 |
| 7.1 | -13915.1 | - | 0 | 8 | 0 | 1 | 45107 | 45116 |
| 7.2 | -13915.2 | - | 0 | 3 | 0 | 0 | 45113 | 45116 |
| 7.3 | -13915.3 | - | 0 | 3 | 0 | 0 | 45113 | 45116 |
| 7.4 | -13915.4 | - | 0 | 3 | 0 | 0 | 45113 | 45116 |
| 7.5 | -13915.5 | - | 0 | 3 | 0 | 0 | 45113 | 45116 |
| 7.6 | -13915.6 | - | 0 | 3 | 0 | 0 | 45113 | 45116 |
| 7.7 | -13915.7 | - | 0 | 3 | 0 | 0 | 45113 | 45116 |
| 7.8 | -13915.8 | - | 0 | 3 | 0 | 0 | 45113 | 45116 |
| 7.9 | -13915.9 | - | 0 | 3 | 0 | 0 | 45113 | 45116 |
| 7.10 | -13915.1 | - | 0 | 3 | 0 | 0 | 45113 | 45116 |
| 7.11 | -13915.1 | - | 0 | 3 | 0 | 0 | 45113 | 45116 |
| 7.12 | -13915.1 | - | 0 | 3 | 0 | 0 | 45113 | 45116 |
| 7.13 | -13915.1 | - | 0 | 3 | 0 | 0 | 45113 | 45116 |
| 7.14 | -13915.1 | - | 0 | 3 | 0 | 0 | 45113 | 45116 |
| 7.15 | -13915.1 | - | 0 | 3 | 0 | 0 | 45113 | 45116 |
| 7.16 | -13915.2 | - | 0 | 3 | 0 | 0 | 45113 | 45116 |
| 7.17 | -13915.2 | - | 0 | 3 | 0 | 0 | 45113 | 45116 |
| 7.18 | -13915.2 | - | 0 | 3 | 0 | 0 | 45113 | 45116 |
| 7.19 | -13915.2 | - | 0 | 3 | 0 | 0 | 45113 | 45116 |
| 7.20 | -13915.2 | - | 0 | 3 | 0 | 0 | 45113 | 45116 |
| 7.21 | -13915.2 | - | 0 | 1 | 0 | 0 | 45115 | 45116 |
| 8 | -13914 | G | 10 | 2 | 45103 | 0 | 0 | 45115 |
| 8.1 | -13914.1 | - | 0 | 0 | 1191 | 0 | 43926 | 45117 |
| 9 | -13913 | T | 1 | 14 | 1 | 45101 | 0 | 45117 |
| 9.1 | -13913.1 | - | 0 | 0 | 0 | 1 | 45116 | 45117 |
| 10 | -13912 | A | 45011 | 0 | 104 | 0 | 3 | 45118 |
| 10.1 | -13912.1 | - | 38 | 0 | 4 | 0 | 45076 | 45118 |
| 10.2 | -13912.2 | - | 0 | 14 | 0 | 0 | 45104 | 45118 |
| 11 | -13911 | G | 15 | 0 | 45103 | 0 | 2 | 45120 |
| 11.1 | -13911.1 | - | 2 | 0 | 0 | 9 | 45109 | 45120 |
| **12** | **-13910** | **C** | **0** | **45101** | **0** | **19** | **0** | **45120** |
| 12.1 | -13910.1 | - | 0 | 0 | 0 | 1 | 45119 | 45120 |
| 13 | -13909 | C | 0 | 45096 | 0 | 23 | 0 | 45119 |
| 14 | -13908 | C | 0 | 45110 | 0 | 10 | 0 | 45120 |
| 15 | -13907 | C | 1 | 45060 | 1 | 37 | 21 | 45120 |
| 15.1 | -13907.1 | - | 0 | 93 | 0 | 0 | 45027 | 45120 |
| 15.2 | -13907.2 | - | 0 | 1 | 5 | 0 | 45114 | 45120 |
| 16 | -13906 | T | 2 | 21 | 1 | 45094 | 2 | 45120 |
| 16.1 | -13906.1 | - | 1 | 18 | 0 | 0 | 45101 | 45120 |
| 17 | -13905 | G | 14 | 1 | 45104 | 1 | 0 | 45120 |
| 18 | -13904 | G | 10 | 0 | 45107 | 2 | 1 | 45120 |
| 18.1 | -13904.1 | - | 0 | 0 | 12 | 3 | 45105 | 45120 |
| 19 | -13903 | C | 0 | 45095 | 0 | 25 | 0 | 45120 |
| 20 | -13902 | C | 0 | 45094 | 0 | 21 | 5 | 45120 |
| 20.1 | -13902.1 | - | 0 | 7 | 0 | 0 | 45113 | 45120 |
| 21 | -13901 | T | 0 | 29 | 1 | 45089 | 1 | 45120 |
| 21.1 | -13901.1 | - | 1 | 0 | 0 | 2 | 45117 | 45120 |
| 22 | -13900 | C | 0 | 45092 | 0 | 27 | 1 | 45120 |
| 22.1 | -13900.1 | - | 0 | 3 | 2 | 0 | 45104 | 45109 |
| 23 | -13899 | A | 45101 | 0 | 8 | 0 | 0 | 45109 |
| 24 | -13898 | A | 45105 | 0 | 3 | 0 | 0 | 45108 |
| 25 | -13897 | A | 45052 | 1 | 39 | 2 | 11 | 45105 |
| 25.1 | -13897.1 | - | 28 | 0 | 0 | 0 | 45043 | 45071 |
| 26 | -13896 | G | 11 | 0 | 45059 | 1 | 0 | 45071 |
| 27 | -13895 | G | 10 | 0 | 45058 | 1 | 1 | 45070 |
| 27.1 | -13895.1 | - | 0 | 0 | 26 | 1 | 45031 | 45058 |
| 28 | -13894 | A | 45051 | 0 | 7 | 0 | 0 | 45058 |
| 29 | -13893 | A | 45042 | 0 | 7 | 1 | 8 | 45058 |
| 29.1 | -13893.1 | - | 13 | 8 | 0 | 0 | 45027 | 45048 |
| 29.2 | -13893.2 | - | 0 | 0 | 0 | 8 | 45039 | 45047 |
| 30 | -13892 | C | 0 | 45034 | 4 | 9 | 0 | 45047 |
| 30.1 | -13892.1 | - | 0 | 0 | 1 | 2 | 45033 | 45036 |
| 31 | -13891 | T | 0 | 2 | 0 | 45034 | 0 | 45036 |
