# Supplementary material for: The Microcephalin Ancestral Allele in a Neanderthal Individual
Source: PLoS One. 2010 May 14;5(5):e10648. doi: 10.1371/journal.pone.0010648 (PMC2871044; doi:10.1371/journal.pone.0010648)
Supplement: Table S3 — Mitochondrial HVR1 variation in the researchers that have been in physical contact with the samples. (0.03 MB DOC) [file pone.0010648.s004.doc]

| **Researcher** | **Task** | **HVR1 Haplotype** |
| --- | --- | --- |
| M.L | Laboratory analysis | 16069,160126,16261,16311 |
| L.M | Laboratory analysis | 16093,16189 |
| C.B | Laboratory analysis | 16209,16223,16292,16311 |
| S.V | Laboratory analysis | 16311 |
| G.C | Laboratory analysis | 16069,16126,16261,16311 |
| L.L | Anthropological study | 16176,16218,16274 |
| S.C | Anthropological study | 16257,16304 |
| D.C | Laboratory analysis | 16193,16278 |
