# Supplementary material for: The Microcephalin Ancestral Allele in a Neanderthal Individual
Source: PLoS One. 2010 May 14;5(5):e10648. doi: 10.1371/journal.pone.0010648 (PMC2871044; doi:10.1371/journal.pone.0010648)
Supplement: Table S4 — Nucleotide misincorporations among clone sequences and 454 reads. The distributions of each possible misincorporation are reported. %P(TsI), %P(TsII) and %P(Tv) refer to the observed frequencies (%) of type I and type II transitions and transversion per site (ignoring indels) and corrected for nucleotide composition. For a given locus, the probability values reported correspond to weight averages over the different amplicons. (0.09 MB DOC) [file pone.0010648.s005.doc]

| **A, Nucleotide misincorporation, Clone sequences** | **L(bp)** | **#Seq.** | **#Sites** | **#PCR** | **AT>GC** | **GC>AT** | **AT>TA** | **CG>GC** | **GC>TA** | **AT>CG** | **Total** | **%GC** | **%AT** | **%P(TsI)** | **%P(TsII)** | **%P(Tv)** |
| --- | --- | --- | --- | --- | --- | --- | --- | --- | --- | --- | --- | --- | --- | --- | --- | --- |
| **Neandertal mtDNA fragments** |  |  |  |  |  |  |  |  |  |  |  |  |  |  |  |  |
| 16,109-16,191 | 81 | 58 | 4698 | 3 | 3 | 9 | 1 | 1 | 0 | 0 | 14 | 42,0% | 58,0% | 0,046 | 0,265 | 0,045 |
| 16,220-16,282 | 63 | 48 | 3024 | 3 | 5 | 4 | 1 | 1 | 0 | 0 | 11 | 44,4% | 55,6% | 0,132 | 0,165 | 0,068 |
| 6,267 | 25 | 36 | 900 | 2 | 0 | 4 | 1 | 0 | 0 | 0 | 5 | 60,0% | 40,0% | 0 | 0,296 | 0,167 |
| **Modern human mtDNA fragments** |  |  |  |  |  |  |  |  |  |  |  |  |  |  |  |  |
| 16,109-16,191 | 81 | 2 | 162 | 1 | 0 | 0 | 0 | 0 | 0 | 0 | 0 | 40,7% | 59,3% | 0 | 0 | 0 |
| 16,220-16,282 | 62 | 12 | 744 | 3 | 0 | 0 | 0 | 0 | 0 | 0 | 0 | 48,4% | 51,6% | 0 | 0 | 0 |
| 6,267 | 25 | 4 | 100 | 1 | 0 | 0 | 0 | 0 | 0 | 0 | 0 | 60,0% | 40,0% | 0 | 0 | 0 |
| **Nuclear loci** |  |  |  |  |  |  |  |  |  |  |  |  |  |  |  |  |
| LCT, ancestral haplotype | 31 | 60 | 1860 | 2 | 0 | 3 | 0 | 0 | 0 | 0 | 3 | 48,4% | 51,6% | 0 | 0,172 | 0 |
| MCPH1, ancestral haplotype | 33 | 153 | 5049 | 4 | 0 | 6 | 1 | 0 | 0 | 0 | 7 | 51,5% | 48,5% | 0 | 0,112 | 0,021 |
|  |  |  |  |  |  |  |  |  |  |  |  |  |  |  |  |  |
| **B, Nucleotide misincorporation, 454 reads** | **L(bp)** | **#Seq.** | **#Sites** | **#PCR** | **AT>GC** | **GC>AT** | **AT>TA** | **CG>GC** | **GC>TA** | **AT>CG** | **Total** | **%GC** | **%AT** | **P(TsI)** | **P(TsII)** | **P(Tv)** |
| **Nuclear loci** |  |  |  |  |  |  |  |  |  |  |  |  |  |  |  |  |
| LCT, ancestral haplotype | 31 | 45102 | 1398162 | 1 | 243 | 233 | 6 | 8 | 7 | 4 | 501 | 48,4% | 51,6% | 0,016 | 0,018 | 0,002 |
| LCT, derived haplotype | 31 | 19 | 589 | 1 | 0 | 0 | 0 | 0 | 0 | 0 | 0 | 45,2% | 54,8% | 0 | 0 | 0 |
| MCPH1, ancestral haplotype | 33 | 12494 | 412302 | 1 | 93 | 202 | 2 | 2 | 12 | 4 | 315 | 51,5% | 48,5% | 0,024 | 0,046 | 0,005 |
| MCPH1, derived haplotype | 33 | 189 | 6237 | 1 | 0 | 0 | 0 | 0 | 1 | 0 | 1 | 51,5% | 48,5% | 0 | 0 | 0,015 |
